# Individualized prognostic calculators in the precision oncology era

**DOI:** 10.18632/oncotarget.26581

**Published:** 2019-01-11

**Authors:** Jeremy M.G. Taylor, Andrew G. Shuman, Lauren J. Beesley

**Affiliations:** Department of Biostatistics, School of Public Health, University of Michigan, Ann Arbor, MI, USA

**Keywords:** calculator, head and neck cancer, nomogram, overall survival, risk prediction

In oncology, predicting outcomes and survivorship for individual patients is critically important. While individualized prognostication is essential, its usefulness depends upon the veracity of the data and the accuracy of the prediction tool.

Hundreds of individualized prognostic calculators (sometimes called nomograms and often available online) have been developed and provide predictions about the likelihood of a future event [1]. These calculators allow care providers or patients to obtain prognostic predictions based on individual clinical characteristics. Unlike staging systems, these calculator outputs are personalized and provide a probability for future events, rather than a treatment recommendation or disease severity classification.

Beesley, et al. [2] evaluated four existing calculators for patients with newly diagnosed oropharyngeal cancer, each designed to predict the 5-year overall survival probability, by applying each calculator to 856 patients in a prospective database. We evaluated (i) whether the predictions from the calculators agree with each other and (ii) whether the calculators provide “valid” and “good” predictions for our population of patients.

To assess calculator quality, we evaluated calibration and discrimination. These measure different aspects of calculator performance, and both are important. About half of the calculators were well calibrated, whereas others tended to provide probabilities that were either too low or too high. There were modest differences in discrimination between the calculators, although some were clearly better than others. The AUC's were in the range 0.74 – 0.80, which is generally considered at the low end of what would be clinically useful.

This paper paralleled previous work evaluating calculators for oral and laryngeal cancers [3, 4]. In these studies, we found poor calibration for the larynx calculators and lower AUC values (0.68-0.74 and 0.65-0.71 for larynx and oral cavity calculators respectively). One major conclusion from all three publications was that predictions from different calculators for the same patient were often noticeably different. For example, it was quite common for the predicted probability to be different by more than 25% for two calculators applied to the same patient. The clinical ramifications of such divergent results are self-evident.

Many of the calculators were previously validated in some way, usually by applying them to another dataset and reporting resulting AUCs. Our data reinforce that a “validated” calculator may not always be a “good” calculator and applicable to all patient populations.

Some calculators include the cancer treatment as an input. While it is clearly desirable to have a model that can suggest a preferred treatment for a specific patient, the task of building such a model is harder than for a prognostic model. In many observational datasets, the choice of treatment is confounded with other factors, which may make resulting predictions based on treatment comparisons less reliable.

The evaluation raises the critical question: What makes a reliable calculator, and how can we make them better? Calculator quality depends on the set of input variables; the quality, size, and timing of derivation data; and the rigor of the statistical approach used to develop the calculator's equations. All these aspects matter. One thing we have learned is that as long the dataset used to develop the calculator is reasonably large, the quality of the data is more important than the size. Criteria for building a good calculator have been published [5].

An obvious way to try to improve calculators is to include more characteristics as input variables. Precision oncology suggests that tumor biology impacts outcomes for each patient, and including important genomic biomarkers should lead to better predictive ability. However, inclusion of additional biomarkers raises formidable issues. Are biomarkers measured in a standardized way, and how many and which need to be included? Larger datasets are going to be needed to construct prognostic models with a large number of input variables. Lastly, even if we can convincingly demonstrate that biomarkers improve the model, the model will not be useful if the biomarkers are not routinely collected in clinical practice.

While some calculators only predict a specific outcome, such as 5-year overall survival, the simultaneous prediction of multiple outcomes (e.g. death, recurrence, and morbidities from treatment) may provide potential improvement. This would also challenge clinicians and researchers to synthesize, present and explain multiple outcome metrics in a manner that health care providers and patients can understand and use in their decision-making.

There is much work to do to improve existing prognostic calculators in oncology. The genomic frontier opens the possibility to better predict outcomes, but also adds complexity in care delivery, model development, and issues of cost and scale. Meeting this challenge will require coordination among researchers, clinicians, statisticians, and social scientists among others to create tools that are not only accurate and reliable, but also can be used broadly and understood widely.
